# Regenerative Medicine

**DOI:** 10.1155/2014/431540

**Published:** 2014-04-09

**Authors:** Ryuichi Morishita

**Affiliations:** Division of Clinical Gene Therapy, Osaka University Graduate School of Medicine, 2-2 Yamada-oka, Suita, Osaka 565-0871, Japan

Regenerative medicine would be the most exciting area in the recent scientific topics. In the history of human gene therapy, the severe combined immunodeficiency caused by the absence of adenosine deaminase (SCID-ADA) was the first monogenic disorder for which gene therapy was developed in 1990. Over 30 patients have been treated worldwide using the current protocols, and recent trials provided the demonstration of long-term clinical efficacy of HSC gene therapy for SCID-ADA in combination with gene and cell therapy. We have now several options, such as gene therapy and/or cell therapy with advanced technology (i.e., cell sheet or drug delivery system), to fight against the severe diseases.

The main focus of this special issue will be on the new and exciting therapy based on stem cell biology, tissue engineering, and gene therapy technology toward regenerative medicine. This special issue contains seven papers, and three papers focus on vascular medicine. J.-I. Kawabe and N. Hasebe described the role of vasa vasorum and vascular stem cells as resident stem cells in atherosclerosis, which might be a novel therapeutic approach for atherosclerosis. M. Shimamura et al. summarized the results of therapeutic angiogenesis in comparison with gene therapy and cell therapy including endothelial progenitor cells. We will reach the final conclusion of therapeutic angiogenesis concept for critical limb ischemia in near future. Y. Saito et al. described the novel findings of therapeutic lymphoangiogenesis which may reduce the edema in patients. Two papers focus on heart regeneration. C. Ikebe and K. Suzuki mainly focused on optimization of cell preparation protocols, especially mesenchymal stem cells, which might be an important process for clinical application. Y. Sawa and S. Miyagawa introduced the experience of their cell sheet-based heart regeneration because their group is the top leader of heart regenerative therapy based on sheet technology. Y. Oie and K. Nishida also show the regenerative medicine for the cornea based on cell sheet technology. They are also top leaders of cornea regeneration therapy based on sheet technology. K. Saga and Y. Kaneda proposed the novel concept of virosome which presents multimodel immunotherapy without viral replication in cancer.

As a result, this special issue includes the topics of atherosclerosis, angiogenesis, and lymphoangiogenesis, heart regeneration, cornea regeneration, and immunotherapy for cancer. We hope that the mixed discussion of these different fields may produce the innovation leading to regenerative medicine in future.

